# Chemotherapy Used to Halt Lower GI Bleeding in a Rare Case of Metastatic Choriocarcinoma to the GI Tract

**DOI:** 10.1155/2016/7620205

**Published:** 2016-09-01

**Authors:** Ralph Kamel, Talal Seoud, Teniola Oluwadamilola, Michael Karass, Emily Grossniklaus, Gabriela Oprea-Ilies, Daniel A. Goldstein, Sanjay Jain

**Affiliations:** ^1^Saint Joseph University, Beirut, Lebanon; ^2^American University of Beirut, Beirut, Lebanon; ^3^Morehouse School of Medicine, Atlanta, GA 30310, USA; ^4^New York Medical College, Valhalla, NY 10595, USA; ^5^Emory University, Atlanta, GA 30322, USA

## Abstract

Choriocarcinoma, a nonseminomatous germ cell tumor, is a rare type of testicular malignancy that tends to occur in young males. It is, however, exceedingly rare for choriocarcinoma to involve the GI tract. In this article, we present a rare case of a 31-year-old male, diagnosed with choriocarcinoma of the left testes, along with several metastases to distant sites. The patient presented with headaches and severe lower GI bleeding due to metastases to the GI tract, which was eventually controlled with systemic chemotherapy, while requiring several units of packed RBCs during his admission to the hospital. An extensive literature review found very few cases of the occurrence of GI bleeding as a consequence of choriocarcinoma due to metastases to the GI tract.

## 1. Introduction

Testicular cancers are a rare type of cancer but are unique in the sense that they are largely curable. In 2014, there were approximately 8,820 new cases of testicular cancer in the USA [1], with peak incidence between 20 and 34 years of age [2]. Testicular carcinoma most commonly presents as a painless testicular mass. However, approximately 20% to 40% of patients with primary testicular cancer present with pain, swelling, hardness of the testicle, or a combination of these symptoms [3].

A type of testicular cancer that is of particular concern is choriocarcinoma, given that it often appears with diffuse metastatic disease and progresses very quickly [2]. Testicular choriocarcinoma is a rare, nonseminomatous germ cell tumor that represents less than 1% of testicular neoplasms and presents as a rapidly growing and aggressive tumor of young males. Notably, the disease is metastatic in nearly half of patients at the time of diagnosis [4]. Choriocarcinomas disseminate via blood and lymphatics with early hematogenous dissemination to lungs, liver, brain, and other visceral sites. Because the average diagnostic delay is 4 to 6 months after symptom onset, patients often present initially with acute disorders resulting from hemorrhage or necrosis of the primary tumors or their metastases [5]. Delayed treatment contributes to increased mortality and morbidity [2]. Although choriocarcinoma was one of the first malignancies for which treatments and complete cure were accomplished with aggressive chemotherapy, the outcomes for metastatic choriocarcinoma remain guarded [2].

In this case report, we will discuss a case of choriocarcinoma in a patient who presented with extensive metastatic disease, notably involving the GI tract. We will review his presentation and imaging, and we will discuss the particular challenges faced when treating this patient.

## 2. Case Presentation

**Figure 1:**
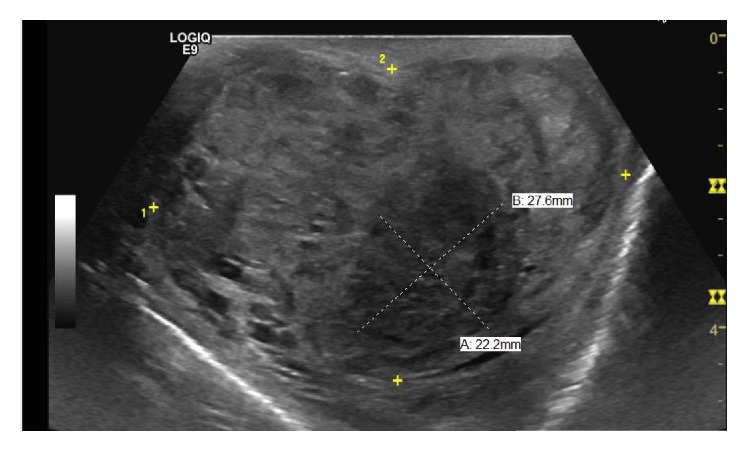
Ultrasound of the L testicle, revealing a heterogeneous infiltrating solid mass.

**Figure 2:**
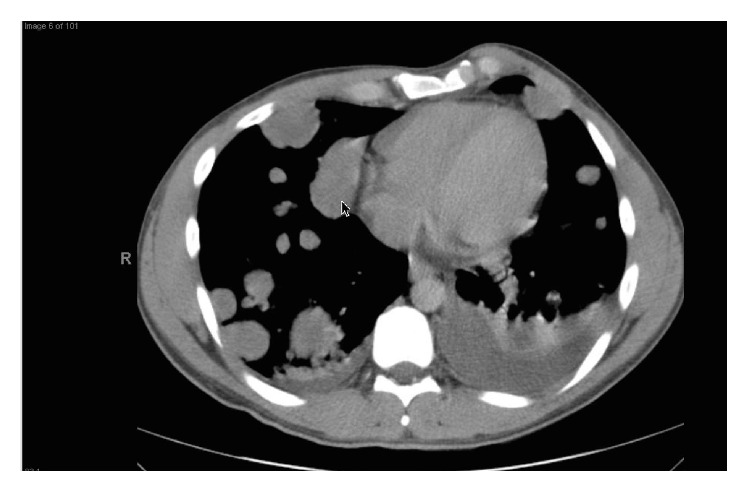
CT of the chest, revealing extensive metastatic disease with numerous pulmonary nodular masses and bilateral pleural effusions.

**Figure 3:**
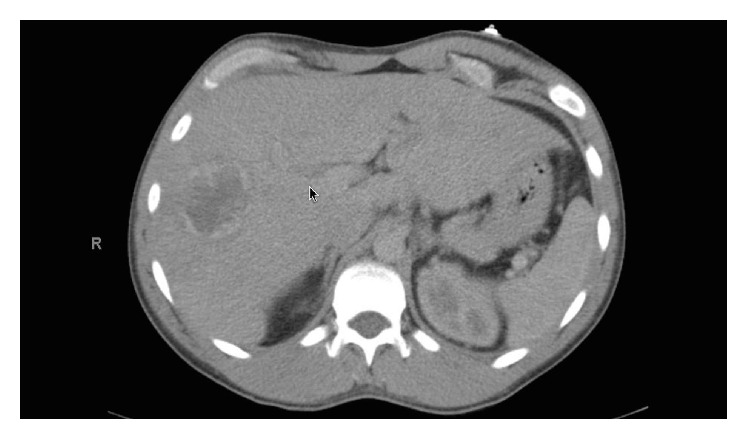
CT of the abdomen, revealing a single hypodense lesion with irregular foci and wall thickening in the right hepatic lobe.

A 31-year-old male with past medical history of asthma presented with headache, melena, and nonbloody nonbilious vomiting, 30 pound (13.6 kg) weight loss, and painful left-sided testicular mass. Patient was found to be anemic with a hemoglobin of 7.0 g/dL (Ref.: 13.5–17.5 g/dL) on admission. Computed Tomography (CT) of head done at the time of admission showed no acute intracranial abnormalities and minimal paranasal sinus disease. Ultrasound (US) of the scrotum showed near completely infiltrated left testicle highly concerning for a malignancy (Figure 1). Due to rising concerns of metastatic disease a CT of chest, abdomen, and pelvis was done. Imaging revealed metastatic lesions to the lungs (Figure 2) and liver (Figure 3) and a left testicular mass. Further investigations revealed that beta-HCG was 217,671 IU (Ref.: 0–3 mIU/mL), Alpha Fetoprotein 2.5 ng/mL (Ref.: <9.0 ng/dL), Estradiol 760 (Ref.: 20–47 pg/mL), and lactate dehydrogenase (LDH) 370 U/L (Ref.: 91–180 U/L). Magnetic Resonance Imaging (MRI) was also done 4 days after admission due to patient's recurrent headaches and was consistent with late subacute lacunar infarct, but there were no findings suggestive of metastatic disease. CT angiogram was subsequently done due to persistent headaches, and this did show metastatic disease involving the subcortical white matter. An echocardiogram was also done that showed likely metastasis in the left ventricle.

The patient subsequently had two endoscopies to evaluate for a GI source of bleeding. He was noted to have duodenitis, while a follow-up colonoscopy was within normal limits. Capsular endoscopy performed during the admission showed actively bleeding lesions in the distal small intestine. Radiology guided embolization of the actively bleeding sites was deemed not to be feasible. A biopsy of lung lesion, in the meantime, confirmed metastatic nonseminomatous testicular cancer.

**Figure 4:**
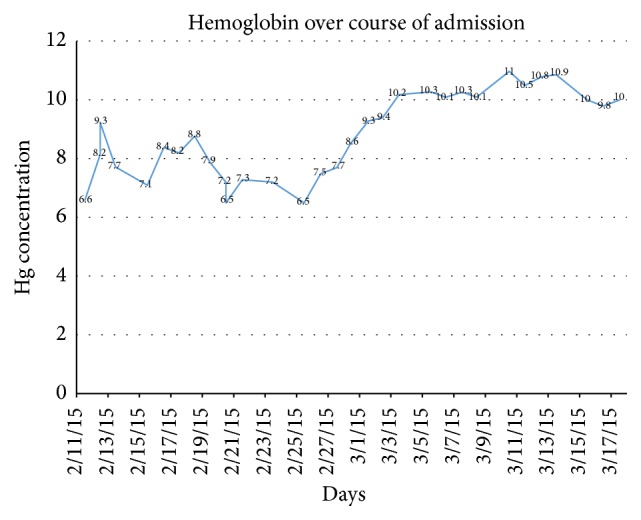
Hemoglobin concentration through patient's admission. The first day of chemotherapy coincides with 2/25. The last day of transfusion was 3/3.

**Figure 5:**
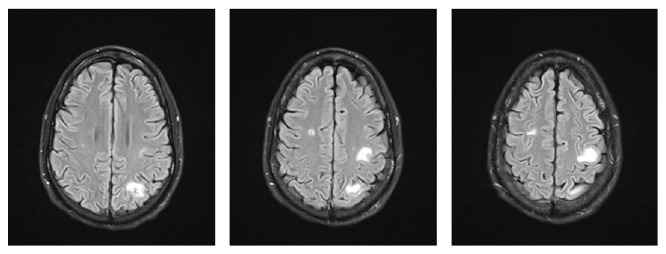
MRI of the brain revealing new interval hemorrhagic and nonhemorrhagic metastases within the bilateral parietal lobes and left occipital lobe visible on T2 FLAIR. This was found after starting chemotherapy with 4 cycles of bleomycin, etoposide, and cisplatin.

The patient was initiated on chemotherapy on day 14 of his admission with cisplatin, bleomycin, and etoposide with the intent to control the bleeding primarily, as well as overall tumor burden. Patient received a total of 18 blood transfusions over a 19-day period. During this time his hemoglobin concentration continued to fluctuate between 6.5 and 11 (Figure 4). He received his last transfusion 14 days prior to discharge and 6 days after start of chemotherapy. His hemoglobin levels remained on average above 10 g/dL several days before discharge, suggesting adequate control of metastases involving the gastrointestinal tract. In addition a repeat MRI two weeks after admission was noted to have interval hemorrhagic and nonhemorrhagic metastases within the bilateral parietal lobes (Figure 5), left occipital lobe, and right lateral eye. Ophthalmology was consulted and recommended outpatient follow-up without the need for immediate interventions. Palliative XRT to the brain, although initially considered, was deferred, since the patient had a relatively small tumor burden involving his brain and was significantly symptomatic from GI bleeding.

At the time of discharge patient had received 1 cycle of chemotherapy and was to receive a total of 4. He recovered from his neutropenia and his pain and headaches were significantly improved. MRI done two months after discharge showed favorable treatment response with no definite new or residual enhancing lesions and his hemoglobin remained above 10 g/dL.

## 3. Discussion

We report the rare clinical presentation of a metastatic testicular choriocarcinoma involving the small bowel, diagnosed after an episode of lower gastrointestinal bleeding and severe headaches. Upon diagnosis, the patient was found to have disseminated disease to the liver, lungs, heart, and brain.

In the setting of testicular choriocarcinoma, metastasis to the gastrointestinal tract is exceedingly rare. Around 0.95% to 5% of the cases present in the small bowel, the duodenum being the most usual site followed by the esophagus, stomach, and colon [6]. Within this group of patients, the most common symptom is hemorrhage presenting as melena or hematemesis. On physical examination, signs of anemia, such as orthostatic hypotension and pale conjunctiva, can be identified in some patients.

In regard to staging and treatment, the patient was diagnosed as having stage IIIC disease, given the presence of visceral metastases involving the liver and brain, and was categorized as a poor-risk patient. The standard chemotherapy regimen for poor-risk patients is 4 cycles of BEP (bleomycin, etoposide, and cisplatin). However, it should be noted that between 20% and 30% of poor-risk patients are not cured with conventional cisplatin-based therapy and less than one-half experience a durable complete response [7].

The patient presented in this case report was admitted with severe gastrointestinal bleeding. Initial surgical management of the intestinal metastatic lesion was deferred because of the identification of multiple sites of metastases, including the lungs and the brain. Systemic chemotherapy was immediately started to control the bleeding while managing the other metastatic foci. Radiation therapy to the brain was also delayed because diffuse metastatic GI disease was considered to be the most life-threatening finding in this patient, as compared to the relatively small secondary brain lesions not accompanied by signs suggestive of increased intracranial pressure and impending herniation (lack of papilledema, no signs of ventricular shifts). The headaches may have been attributable to evolving metastatic disease, as evidenced by a subsequent MRI, while the nausea and vomiting were likely secondary to metastatic involvement of the gastrointestinal tract. The GI bleeding ceased during the first cycle of chemotherapy, an important clinical course adding to the efficacy of chemotherapy in managing systemic disease as well as acute complications of bowel metastases.

The patient was closely monitored for the early detection of treatment complications. The start of chemotherapy can complicate the course of the disease by the development of a very rare, life-threatening syndrome called the choriocarcinoma syndrome, which is characterized by hemorrhage into the metastatic sites accompanied by a significant rise in beta-HCG levels [4]. Given the extent of the disease spread and its aggressive nature, tumor lysis syndrome was a concern as documented cases in the literature were found. However, in this patient, no laboratory or clinical evidence of tumor lysis syndrome was noted. Given the cardiac involvement in this patient, the team was concerned about the development of life-threatening cardiac arrhythmias and embolic cerebral ischemia; however, these complications were luckily avoided during the first round of chemotherapy. The only neurologic symptom that was noted was severe headache that was attributed to the brain metastases and no focal deficits appeared during his hospital stay.

In conclusion, our patient was diagnosed with diffuse metastatic testicular choriocarcinoma that presented with acute gastrointestinal hemorrhage complicated by anemia. However, the therapeutic priority was to address the disseminated disease rather than the localized secondary small bowel lesions or that of his brain disease. The patient was treated with transfusions of packed red blood cells (pRBCs) and the chemotherapy proved efficient in controlling the gastrointestinal hemorrhage while controlling extraintestinal metastatic disease.

This case highlights the importance of considering the rare possibility of a metastatic testicular cancer in a young male patient presenting with acute gastrointestinal bleeding. The management should be based on aggressive chemotherapy with a curative intent.
